# Monitoring of Pediatric Type 1 Diabetes

**DOI:** 10.3389/fendo.2020.00128

**Published:** 2020-03-17

**Authors:** Brynn E. Marks, Joseph I. Wolfsdorf

**Affiliations:** ^1^Division of Endocrinology and Diabetes, Children's National Hospital, Washington, DC, United States; ^2^Division of Endocrinology, Boston Children's Hospital, Boston, MA, United States

**Keywords:** type 1 diabetes (T1D), diabetes management, blood glucose self monitoring, continuous glucose monitor, ketone

## Abstract

Regular self-monitoring of blood glucose levels, and ketones when indicated, is an essential component of type 1 diabetes (T1D) management. Although fingerstick blood glucose monitoring has been the standard of care for decades, ongoing rapid technological developments have resulted in increasingly widespread use of continuous glucose monitoring (CGM). This article reviews recommendations for self-monitoring of glucose and ketones in pediatric T1D with particular emphasis on CGM and factors that impact the accuracy and real-world use of this technology.

## Introduction

Frequent blood glucose (BG) monitoring is a cornerstone of intensive diabetes management and is associated with lower hemoglobin A1c (A1c) values and decreases the occurrence of both hypo- and hyperglycemia (1–3). Improved glycemic control, as measured by A1c, is associated with decreased frequency of diabetic ketoacidosis and severe hypoglycemia, and decreased risk of long-term complications, including microvascular disease, neuropathy, and neurocognitive deficits (2, 4).

Self-monitoring of blood glucose (SMBG) levels became the standard of care for type 1 diabetes (T1D) after the development of the first glucose meter for home use in 1970. The size, speed and accuracy of glucose meters have improved over time, and the volume of blood required for testing has decreased substantially. Despite these improvements in performance characteristics of glucose meters, little changed in home self-monitoring strategies until the development of the first real-time continuous glucose monitor (CGM) in 1999. Since then, the accuracy of CGM has improved considerably such that commercially available systems now allow patients to make treatment decisions based on sensor glucose values alone without requiring a confirmatory fingerstick BG value.

More recently, integration of CGM with continuous subcutaneous insulin infusion (CSII) devices (insulin pumps) has led to the development of algorithm-controlled pumps that suspend insulin delivery when a low glucose level is predicted to occur within the ensuing 30 min, as well as hybrid closed loop systems that can both suspend insulin delivery to prevent hypoglycemia and automatically administer additional insulin to correct hyperglycemia. Use of these devices for management of T1D is rapidly becoming the standard of care. However, these devices are unfortunately not universally available largely for economic reasons. It is also important to understand that CGM devices are not completely reliable at the present time as CGM sensor glucose readings can be inaccurate for a variety of reasons. Despite decreasing day-to-day reliance on fingerstick BG monitoring for increasing numbers of patients throughout the world who are now routinely using CGM, SMBG, and urine and blood ketone monitoring continue to be important tools for the management of T1D. This article examines the roles of fingerstick BG monitoring, CGM, and urine and blood ketone monitoring in pediatric T1D management.

## Discussion

### Blood Glucose Monitoring

The ability to accurately measure BG on a drop of blood obtained by lancing a fingertip is arguably the most important advance in diabetes management since the discovery of insulin. Frequent, accurate fingerstick BG monitoring (BGM) performed by the patient or care provider has, until the advent of CGM, been the cornerstone of intensive management of T1D in children and adolescents, and will continue to serve this purpose for patients who do not have access to CGM (5, 6). SMBG enables patients and families to determine the current BG concentration and to measure levels at various times throughout the day. Every child should have an accurate glucose meter and enough test strips to be able to measure BG levels with sufficient frequency to optimize glycemic control (5). The International Society for Pediatric and Adolescent Diabetes (ISPAD) and American Diabetes Association (ADA) recommendations for glycemic targets are outlined in Table 1 (6, 7).

**Table 1:** ISPAD and ADA glycemic and A1c targets (6, 7).

	**ADA**	**ISPAD**
A1c target	<7.5%	<7.0%
Pre-meal	90–130 mg/dL	70–130 mg/dL
Postmeal	n/a	90–180 mg/dL
Before bedtime	90–150 mg/dL	90–150 mg/dL

*ADA, American Diabetes Association; ISPAD, International Society for Pediatric and Adolescent Diabetes*.

The results of SMBG inform decisions about immediate rapid-acting insulin doses, planning before and throughout exercise, and the treatment of hypoglycemia. The identification of patterns and trends through regular review of recent data also informs decisions about adjustments to doses of basal insulin, insulin-to-carbohydrate ratios, and insulin sensitivity or correction factors. BGM may need to be performed up to 6–10 times per day to identify patterns or trends in order to adjust the insulin regimen, perform “real-time” correction of hyperglycemia, evaluate the impact of foods on postprandial glycemia, and confirm and treat hypoglycemia.

For successful implementation of an intensive diabetes regimen, the four most essential BG measurements are performed before each meal and at bedtime. Premeal measurements are needed to determine the dose of insulin for meals and to guide adjustments for physical activity in order to achieve the patient's target BG level. These measurements are also used to determine patterns of glycemia over time, which guide adjustments of the regimen. BGM must be accompanied by regular data review to allow for appropriate insulin dose adjustments to optimize glycemic control. Before making a change in the regimen, one should examine the BG pattern over a period of at least 3–5 days seeking to identify a consistent pattern of BG levels outside the target range. The bedtime BG measurement is used to assess the adequacy of the dinnertime dose of insulin and to inform decisions aimed at preventing nocturnal hypoglycemia. The value obtained in the morning before breakfast is used to assess overnight glycemic control.

Frequent BGM allows patients to promptly correct glucose values that are above target range, thereby minimizing exposure to hyperglycemia. Assessment of postprandial glycemia 2–3 h after a meal is necessary to optimize insulin doses for meal-time coverage of carbohydrates (as well as protein and fat). Also, periodic BG measurements between midnight and 4 a.m. are valuable to detect unrecognized nocturnal hypoglycemia, and are especially valuable after strenuous physical activity during the day. Nocturnal BG measurements should be performed more frequently when the basal insulin dose is being adjusted. For safety, BGM should routinely be performed to confirm symptoms of hypoglycemia and before driving or operating hazardous machinery. SMBG before, during, and after exercise, along with appropriate modifications of both basal and bolus insulin dosing and consumption of additional carbohydrates during and after exercise, enables patients with T1D to safely perform strenuous competitive exercise and decreases the risk of severe hypoglycemia (8). These recommendations are particularly relevant for swimming owing to the additional safety concerns and potential difficulty rescuing a person from and treating an episode of severe hypoglycemia in the water. Table 2 shows recommendations regarding the timing and frequency of BGM.

**Table 2:** Recommendations for blood glucose monitoring.

**When should BG be measured**
•Routinely before meals and snacks. • Intermittently 2–3 h after meals to evaluate meal-time doses of rapid-acting insulin. • Before, every 1 h during, and after strenuous exercise. • At bedtime and before breakfast to assess the action of basal insulin. • Periodically overnight between 12 a.m. and 4 a.m., especially after strenuous exercise, and when adjusting basal insulin doses. • Before driving a car or operating potentially hazardous machinery. • To confirm symptoms of hypoglycemia and monitor response to treatment. • During intercurrent illness (together with ketone monitoring, see below).

Numerous single center and population studies have shown a relationship between a greater frequency of SMBG and lower A1c concentration as early as 1-year after diagnosis of T1D (9) as well as in patients with established diabetes of longer duration (1–3, 10–13).

### Glucose Meters

Patients should receive proper training in the technical aspects of SMBG as accurate measurement of BG values is essential for correct therapeutic decisions (14, 15). Numerous glucose meters are commercially available and their ease of use and accuracy have increased considerably over time. Most meters do not require manual input of the batch-specific code or calibration, are easier to use, and the required volume of blood has decreased to ~0.3–1 μL. It is desirable to use a device that can be downloaded at home and in the clinic or physician's office to enable review of the BG data. Some glucose meters are Bluetooth enabled; these systems automatically sync glucose meter data to smartphones, websites, and apps, which can facilitate data review for patients at home.

The most recent published requirements for meter accuracy are defined by the International Organization for Standardization (ISO) in the standard EN ISO 15197:2013 (*in vitro* Diagnostic Test Systems—Requirements for Blood Glucose monitoring Systems for Self-Testing in managing Diabetes Mellitus. EN ISO 15197:2013). The US Food and Drug Administration (FDA) does not use this standard as part of the clearance process for these devices. In 2016, the FDA developed its own standard for BG meters for over-the-counter use that was similar to ISO 15197:2013. In defining an acceptable level of accuracy, these two standards both require 95% of data pairs between a BG meter measurement and a reference measurement to be within 15% for BG values >100 mg/dL (5.6 mmol/L). For BG values <100 mg/dL (5.6 mmol/L), ISO 15197:2013 requires data pairs to be within 15 mg/dL (0.8 mmol/L), whereas the more stringent FDA requirement is for data pairs to be within 15%.

A recent assessment of the accuracy of 18 different commercially available BG meters for personal use showed that their accuracy varied considerably, and several BG meters did not meet a predefined accuracy standard (16). In order to be compliant with regulatory standards in this study BG results had to be within 15% of a reference plasma glucose value ≥100 mg/dL or within 15 mg/dL for plasma glucose <100 mg/dL (5.6 mmol/L) (16). Only 6 of 18 best-selling personal BG meters met a protocol-specified accuracy standard similar to current ISO and FDA standards on three of three studies. Whenever possible, therefore, an accurate meter [especially in the low glucose range <70 mg/dL (3.9 mmol/L)] that meets these standards should be prescribed for children with T1D.

### Inaccuracies in BGM

The impact of physiologic and environmental factors on the accuracy of BGM depends on the enzymatic reaction the device uses to measure glucose concentrations. Glucose oxidase (GO) and glucose dehydrogenase (GDH) are the two most commonly used enzymes. Reactions catalyzed by GO are sensitive to alterations in the partial pressure of oxygen. This enzyme has a high specificity for glucose and is therefore not affected by other sugars. GDH-based reactions are catalyzed by different cofactors, most notably pyrroloquinolinequinone (GDH-PQQ). Because the reaction catalyzed by this cofactor has a lower specificity for glucose, cross-reactivity occurs with maltose, galactose, lactose, and xylose. Since the discovery of this interferent, many manufacturers have changed the enzyme to mutant GDH-PQQ which is not affected by maltose.

The accuracy and reliability of SMBG results depend on the proficiency of users in performing the measurements. It is therefore essential that patients and care providers receive thorough education and training in the proper use of their specific devices (14, 15). Children who are able to independently perform SMBG must be properly supervised because it is not unusual for children to fabricate data, either intentionally or accidentally, with potentially disastrous consequences. Many operator related factors can affect BG measurement results, including: failure to handle the system according to the manufacturer's instructions, the use of deteriorated (expired) test strips, incorrect storage of strips, or the presence of traces of glucose on the fingertips, such as from touching fruit or candy. Extreme temperatures and rapid changes in ambient temperature, such as moving from outdoors to indoors during the winter, can also impact the accuracy of SMBG (17).

Several other physiologic factors are known to impact the accuracy of BGM readings. Causes of falsely high BGM values include: low hematocrit (<35%), hyperuricemia, low partial pressure of oxygen (<45mm Hg) in GO based devices, and acetaminophen. Factors that falsely decrease BGM values include: elevated hematocrit (>45%), hypertriglyceridemia, decreased tissue perfusion at the testing site, elevated partial pressure of oxygen (>150mm Hg) in GO-based devices, and ascorbic acid (18–24). High maltose levels in peritoneal dialysis solution, intravenous immunoglobulin G, Rho (D) immunoglobulin, Abatacept, and tositumomab cause falsely high BG concentrations with glucose meters that use GDH-PQQ (25). When patients are receiving medications with high levels of maltose, we suggest that clinicians review the user manual to ascertain the specific enzyme in the test strips being used. See Table 3 and the article by Schmid et al. for further details about the impact of user error, ambient conditions, and physiologic factors on BGM accuracy (15).

**Table 3:** Factors impacting the accuracy of self-monitored blood glucose.

	**Falsely low BG results**	**Falsely high BG results**	**Variable impact on accuracy**
**USER ERROR**			
Improper handwashing	• Residual water on fingertips	• Traces of glucose-containing substances on fingertips • Residual alcohol on fingertips	
Deteriorated test-strips			• Improper coding • Improper storage • Expired test-strips
**AMBIENT CONDITIONS**			
Altitude			• High Altitude (>2,000 m)
Oxygen concentrations	• High partial pressure of oxygen	• Low partial pressure of oxygen	
Extreme temperatures	• Cold temperatures (<10°C) • Rapid decrease in ambient temperature	• Rapid increase in ambient temperature	• Hot temperatures (>39°C)
Alternative test sites	• Testing at cold sites (e.g., forearm)		• Lag time created by decreased blood flow to alternative sites
**PHYSIOLOGIC CONDITIONS AND MEDICATIONS**			
Laboratory values	• Elevated hematocrit (>45%) • Hypertriglyceridemia	• Low hematocrit (<35%) • Hyperuricemia	
Altered tissue oxygenation	• Partial pressure of oxygen >150 mmHg^*^ • Poor perfusion at testing site	• Partial pressure of oxygen <45 mmHg^*^	
Medication related	• Ascorbic acid	• Acetaminophen • Maltose (in GDH-PQQ based devices only)	

* *Glucose oxidase-based devices only*.

It is important for patients to know that test strips contain a complex enzymatic reaction layer and should always be stored in their original vials, tightly closed, in order to preserve their analytical stability, as test strips in open vials deteriorate more rapidly. Environmental and sampling conditions such as high altitude, partial pressure of oxygen (in GO-based systems), ambient temperature, and the use of alternative test sites also can influence results. In order to obtain accurate and reliable data, the device must be properly maintained and cleaned. The patient must use proper technique, including washing and completely drying hands, or cleaning the skin with an alcohol swab and allowing time for the alcohol to dry before lancing the skin. Patients/parents of children with T1D must receive comprehensive education not only about how to perform SMBG but also how to interpret the results. When they change their BGM system, proper use of the new device should be reviewed by a healthcare professional.

### Recording BG Data

BG data should be recorded using a logbook, spreadsheet, smart meter, app, or a cloud-based program that enables patients to record and review BG values, insulin doses, exercise, and amount of carbohydrate consumed. Successful intensive diabetes management requires active engagement of the patient/family and regular review of the data, as well as sharing the data with the diabetes care team at the time of in-person consultation or between clinic visits, especially when BG values are persistently out of the target range. Patients/parents must be taught how to use the data to assess the efficacy of therapy and, ideally, to self-adjust the components of their treatment regimen to achieve individual BG goals. Most glucose meters have an electronic memory that enables data to be downloaded to a computer. Irrespective of whether the data are recorded manually in a logbook or electronically, it is valuable for patients/parents to examine the data for patterns and trends in the intervals between visits with their diabetes care providers, so that adjustments can be made when necessary. Guidance from the care team is particularly important for patients with recent onset diabetes. It is sobering to note that despite having the key to improved glycemic control literally “at their fingertips,” a report from the T1D Exchange registry in the US showed that nearly two-thirds of patients/families *never* download their SMBG data (26).

While SMBG was the standard of care in pediatric T1D management for decades, the widespread use of CGM is anticipated to become the new standard of care within the next few years.

### Continuous Glucose Monitoring

CGMs are minimally invasive devices that use a subcutaneous sensor to measure changes in interstitial glucose values (27). CGMs consist of three essential components: a sensor that detects the changes in glucose, a transmitter which relays the signal from the sensor to a receiver, and a receiver which uses an algorithm to convert the signal into a glucose value that is displayed on the device. Because sensors are continuously attached to the skin, after a brief warm up period, CGM systems report and record the sensor glucose values every 5–15 min. While some CGM systems require the user to enter SMBG data to calibrate the CGM once every 12 h, other systems are factory calibrated and do not require user calibration.

It is important to appreciate that CGM measures interstitial glucose rather than BG concentrations. The differences in glucose levels between these two different compartments in the body introduces a concept referred to as lag time. Interstitial glucose levels are determined by the flow of glucose according to the concentration gradient between the vascular space and the interstitial space. The physiologic lag time created by the concentration gradient leads to discrepancies between sensor glucose values and fingerstick BG values. When BG levels are not changing rapidly, there is minimal physiologic lag time and therefore no significant difference in the glucose concentration between blood and interstitial fluid. Lag time is most pronounced when BG levels are changing rapidly. When BG levels are rising rapidly, sensor glucose values will be falsely low for a period of time. Conversely, when BG values are rapidly falling, sensor glucose values will be falsely high for a period of time. If patients are unaware of this phenomenon, the lag time in sensor glucose readings when blood glucose levels are decreasing rapidly may result in failure to promptly treat hypoglycemia. After treating hypoglycemia, the lag time may lead to prolonged sensor alerts for hypoglycemia, which may result in over-treatment.

The lag time between blood and sensor glucose values varies depending on individual patient factors, fasted vs. fed state, and activity levels (28–30). In addition to physiologic lag time, additional delays occur due to the time required for the sensor reaction to detect changes in interstitial glucose concentrations and for signal processing (31, 32). There is sparse literature exploring the total duration of lag time. One carefully conducted study using radiolabeled glucose isotopes identified a mean lag time of 6 min with a maximum of 10 min for the isotope to appear in the interstitial fluid after intravenous injection (29). Analysis of real-world use of these systems under varied conditions have found a total lag time between blood and sensor glucose values ranging from 5 to 40 min depending on the clinical circumstances (30, 32). Clinicians and patients must account for lag time when making treatment decisions based on CGM sensor glucose values to avoid over treatment of hypo- and hyperglycemia.

The ability to detect not only the concentration of glucose in the interstitial tissue, but also changes over time, allows for CGMs to report a numerical sensor glucose value along with the rate of change. The rate of glucose change is displayed on the receiver using arrows. Each CGM system has its own unique arrow system to convey specific rates of change of sensor glucose levels (Table 4). The rate of change conveyed by the trend arrows and predicted glucose in 30 min can be used to overcome lag-time and to optimize clinical decision-making and insulin dosing (33–35).

**Table 4:** Rates of sensor glucose change as indicated by different CGM systems.

**Rate of sensor glucose change**	**Dexcom**	**Medtronic**	**FreeStyle Libre**	**Eversense**
0–1 mg/dL/min	→	No arrow	→	→
1–2 mg/dL/min	↗ or ↘	↑ or ↓	↗ or ↘	↗ or ↘
2–3 mg/dL/min	↑ or ↓	↑↑ or ↓↓	↑ or ↓	↑ or ↓
> 3 mg/dL/min	↑↑ or ↓↓	↑↑↑ or ↓↓↓	n/a	n/a

### Types of CGM Systems

There are three main types of CGM systems and each conveys sensor glucose information to the user in a different manner: blinded, real-time, and intermittently scanned or flash CGM (isCGM). Blinded CGMs do not provide the user with real-time data, but are downloaded and reviewed retrospectively. Blinded CGM typically is applied by a healthcare provider and used for a short period of time to gain insight into glycemic trends. While blinded CGMs may provide insight into glycemic excursions not captured by fingerstick BGM and yield short-term improvements in glycemic control, there is no evidence they have a sustained long-term benefit (36, 37). The remainder of this section will specifically address the benefits of using real-time and isCGM.

Real-time CGMs provide the user with the interstitial glucose values as they become available. These devices include alarms for hypo- and hyperglycemia, rapid rates of glycemic change, and predicted hypo- and hyperglycemia. Users can customize these alarms to their specific needs and preferences. Real-time CGM systems can use a device-specific receiver or a smart device to display sensor glucose values. Smart devices, but not device-specific receivers, send data to the cloud so that followers remote from the individual using the CGM can track glycemic trends in real time on their smart devices. Examples of real-time CGM systems include: Dexcom (San Diego, CA), Medtronic Guardian (Medtronic, Northridge, CA), and Eversense (Senseonics, Germantown, MD).

isCGM systems only display sensor glucose values when the user scans the sensor and do not provide users with alarms for glycemic excursions out of the defined target range. Freestyle Libre (Abbott Diabetes Care Inc., Alameda, CA) is the only isCGM system currently available. The Libre has an 8-h memory; if the sensor is not scanned at least once every 8-h, thereby transferring sensor glucose data from the sensor to the receiver, the data will be permanently lost. Once the data have been transferred to the receiver it is retained for 90-days. Although not approved by regulatory agencies, some isCGM users purchase a device worn over the top of the Libre sensor (MiaoMiao Smart Reader) that transforms it into a real-time CGM, automatically sending data from the sensor to a smart phone without the need to scan the sensor. Table 5 shows an overview of the currently available CGM systems.

**Table 5:** Comparison of currently available CGM systems.

	**Dexcom G6**	**Medtronic Guardian 3**	**FreeStyle Libre**	**Eversense**
**Calibration**	**Not required, can perform**	**Every 12 h**	**Not required, cannot perform**	**Every 12 h**
Non-adjunctive dosing indication	Yes	No	Yes	Yes
MARD	9.0%	9.6% (varies by site and calibration frequency)	9.4%	8.5%
Alerts	Yes	Yes	No	Yes
Sensor warm up time	2 h	2 h	1 h	~26 h
Sensor wear time	10 days	7 days	14 days	90 days
Transmitter life	90 days	1 year, rechargeable	14 days	1 year, rechargeable
Share/follow app	Yes	Yes	Yes	Yes
Interfering substances	No acetaminophen interference (up to 4 g/day)	Acetaminophen	Vitamin C, Aspirin	Mannitol, Tetracycline, Aspirin
Unique features	T:slim integration for Basal IQ and Control IQ	Sugar IQ decision support	No alerts for highs/lows unless scanned; Loses data if not scanned every 8 h	Implanted CGM; On body vibration alerts without receiver

*Non-adjunctive dosing refers to the ability to make treatment decisions for both hypo- and hyperglycemia based on a CGM sensor glucose value without a confirmatory fingerstick BG measurement*.

*MARD or mean absolute relative difference is the average of the absolute differences between reference blood glucose measurements and glucose measurements obtained by CGM*.

### Unique Features of CGM

#### Alerts and Alarms

Because real-time CGM systems sense glucose levels and detect glucose trends, these systems have the ability to alert patients to both actual and impending hypoglycemia. The Medtronic Guardian, Eversense, and Dexcom systems allow users to customize alerts for hypoglycemia and hyperglycemia at different times of day. These systems also afford users the option to receive alerts when sensor glucose levels are rising or falling rapidly. All alerts can be turned off by the user except for the urgent low alert (at a sensor glucose of 55 mg/dL on the Medtronic and Dexcom systems) and a customizable low alert on the Eversense system.

While alerts and alarms can help to improve glycemic control, their impact on day-to day-life must be carefully considered. Alarm fatigue is one of the factors most frequently cited by patients who discontinue CGM use (38–40). Alarm settings must be appropriately individualized for each patient to optimize glycemic control while minimizing the impact on quality of life, taking into consideration age, current glucose control, impact on daily life, and hypoglycemia awareness.

#### Real Time Adjustment for Trend Arrows

Lag time of CGM systems and the ability to sense glucose trends can be incorporated into insulin dosing decisions in an effort to optimize glycemic control. At the present time, both Dexcom G5 and G6, Freestyle Libre, and Eversense have regulatory approval allowing diabetes treatment decisions to be made without a confirmatory fingerstick BGM. Guidelines for insulin adjustments based on the patient's correction factor (CF) and the trend arrows have been published for both the Dexcom and Freestyle Libre (33–35). System-specific guidelines were needed, unfortunately, because of the different rates of change conveyed by the trend arrows for each system (Table 4). Both pediatric and adult guidelines have been published for Dexcom CGM (Table 6). Only adult guidelines exist for the Freestyle Libre because it is not approved for use in patients under 18 years of age. The recommended insulin dose adjustments are based on the trend arrow and the predicted glucose in 30 min. As shown in Table 6, recommended insulin dose adjustments for any given CF are more aggressive for adult patients than pediatric patients because of greater glycemic variation characteristic of pediatric patients with T1D (41).

**Table 6:** Pediatric and adult insulin dose adjustments for trend arrows for Dexcom G5 and G6 and Freestyle Libre [adapted from Laffel et al. (33), Aleppo et al. (34), and Kudva et al. (35)].

	**Dexcom G5/G6 insulin dose adjustments**				**Freestyle Libre insulin** **dose adjustments**	
	**Pediatric recommendations**		**Adult recommendations**		**Adult recommendations**	
	**Correction factor (mg/dL)**	**Insulin dose adjustment**	**Correction factor (mg/dL)**	**Insulin dose adjustment**	**Correction factor (mg/dL)**	**Insulin dose adjustment**
↑↑ or ↓↓	<25 25 to <50 50 to <75 75 to <125 ≥125	±4.0 units ±3.0 units ±2.0 units ±1.0 units ±0.5 units	<25 25 to <50 50 to <75 ≥75	±4.5 units ±3.5 units ±2.5 units ±1.5 units		
↑ or ↓	<25 25 to <50 50 to <75 75 to <125 ≥125	±3.0 units ±2.0 units ±1.0 units ±0.5 units No adjustment	<25 25 to <50 50 to <75 ≥75	±3.5 units ±2.5 units ±1.5 units ±1.0 units	<25 25 to <50 50 to <75 ≥75	±3.5 units ±2.5 units ±1.5 units ±1.0 units
↗ or ↘	<25 25 to <50 50 to <75 75 to <125 ≥125	±2.0 units ±1.0 units ±0.5 units No adjustment No adjustment	<25 25 to <50 50 to <75 ≥75	±2.5 units ±1.5 units ±1.0 units ±0.5 units	<25 25 to <50 50 to <75 ≥75	±2.5 units ±1.5 units ±1.0 units ±0.5 units
→	All CF	No adjustment	All CF	No adjustment	All CF	No adjustment

*CF, correction factor*.

The recommended insulin dose adjustments based on trend arrows should be regarded as a starting point and adjustments should be individualized based on prior responses (33). The authors recommend waiting at least 3 h from the last bolus before applying these insulin dose adjustments based on trend arrows and also suggest avoiding application of these “rules” at bedtime and during periods of intercurrent illness, increased activity, and ketosis.

Although no official recommendations for this approach have been published, others have advocated a more user-friendly approach for Dexcom G5 and G6 users. The so-called “30-60-90 rule” suggests adding 30 mg/dL (1.7 mmol/L) to the present sensor glucose value when the sensor glucose is changing by 1–2 mg/dL/min (0.06–0.1 mmol/L/min), 60 mg/dL (3.3 mmol/L) when the sensor glucose is changing by 2–3 mg/dL/min (0.1–0.2 mmol/L/min), and 90 mg/dL (5.0 mmol/L) when the sensor glucose is changing by > 3 mg/dL/min (>0.2 mmol/L/min). This approach uses the predicted sensor glucose in 30 min based on the rate of change conveyed by the trend arrow. For example, with a ↑ arrow on the Dexcom CGM, the sensor glucose is rising by 2–3 mg/dL/min (0.1–0.2 mmol/L/min) and is predicted to be 60–90 mg/dL (3.3–5.0 mmol/L) higher in 30 min. Thus, a conservative adjustment would be to add 60 mg/dL (3.3 mmol/L) to the current sensor glucose value when calculating the insulin dose to correct hyperglycemia.

#### Data Sharing

All of the currently available real-time and isCGM systems offer the opportunity to share data. This feature allows those who use a smart phone as a CGM receiver to share their data with remote followers. The user's data are uploaded from the smart device to the cloud and then sent to followers either via smartphone apps, websites, or text messages, depending on the platform. Users should be aware that CGM sharing may lead to significant smart phone data usage.

Data on the impact of CGM sharing are mixed. Recent studies found that nearly 95% of pediatric Dexcom users share their data with at least one follower, with equal frequency of data sharing among patients ages 2–5 years, 6–12, and 13–18 years (42). Although the authors reported decreased mean glucose values and increased time in range for those who use the share function, the effects were minor. Qualitative analysis of online blogs suggests that data sharing enhances feelings of safety, but also emphasizes the importance of setting boundaries and avoiding judgments about glycemic excursions (43). The importance of setting communication boundaries with parents and caretakers, particularly during adolescence, should focus on enhancing patient safety and must avoid “policing” teen behavior in order to minimize conflict, which has been cited as a factor discouraging CGM use among adolescents (40, 44).

#### Implantable CGM

At the present time, the Eversense CGM is approved for patients ≥ 18 years of age; however there are ongoing studies aimed at seeking approval for patients as young as 6 years. Unlike other CGM devices wherein the sensor is inserted percutaneously, the Eversense CGM is surgically implanted in the upper arm (45). The transmitter sits on top of the skin, overlying the implanted sensor, and has a larger profile than other CGM devices on the market. Sensors are approved to remain in place for up to 90 days in the United States and 180 days in Europe; Eversense is seeking approval to extend wear time up to 360 days. While the Eversense CGM has exceptional accuracy, particularly when BG is <70 mg/dL, and can provide users with vibratory alerts from the transmitter in the absence of a nearby phone receiver (unlike other CGMs), uptake of this technology has been slow.

#### Automated Insulin Delivery

Although not the specific focus of this article, CGM is an essential component of threshold suspend, predictive low glucose suspend, and hybrid closed loop insulin delivery systems. Studies have clearly shown marked improvements in glycemic control while also reducing hypoglycemia in patients using these algorithm-driven automated insulin delivery systems (46, 47).

### CGM Accuracy

While the ISO is universally accepted as an accuracy standard for BGM, no such standard exists for CGMs. The most commonly used metric to assess CGM accuracy is the mean absolute relative difference (MARD). MARD is the average of the absolute difference between a reference measurement and CGM measurement; a lower MARD value indicates a more accurate sensor. In 1999, the first commercial CGM system had a MARD of 26% (48), whereas the MARD of currently available sensors ranges from 9.0% without any calibration for the Dexcom G6 and 9.4% for the Freestyle Libre, to 9.6% for the Medtronic Guardian with three to four daily calibrations (30, 49–51). It should be noted, however, that the MARD value is impacted by many factors beyond the accuracy of the sensor, including: the glucose concentration, the absolute number of data points, the rate of glucose change, intensive exercise, and missing data points (52–54). Therefore, different studies have resulted in different MARD values for the same device (55). To date, head-to-head comparison of the accuracy of BGM and CGM systems has been limited due to differences in how these systems are assessed for accuracy (56) and at the present time there is no universally accepted protocol to compare the different devices to assess their accuracy.

The creation of the “integrated CGM” or iCGM distinction by the US Food and Drug Administration (FDA) in 2018 allowed for an approved CGM system to be used as part of an integrated system with other compatible medical devices and electronic interfaces. The increased demand for CGM accuracy and transparency required to receive this distinction may also pave the way for better CGM assessments in the future (56, 57). Table 7 shows a comparison of the ISO and iCGM criteria.

**Table 7:** Comparisons of the accuracy requirements for blood glucose meters, as dictated by the ISO 15197:2013, and iCGM, as required by the US FDA.

	**ISO 15197:2013**	**FDA iCGM**
Overall Accuracy	≥95% within ± 15 mg/dL for glucose concentrations <100 mg/dL ≥95% within ± 15% for glucose concentrations >100 mg/dL	>87% within ± 20%
**ACCURACY REQUIREMENTS BY GLUCOSE CONCENTRATION**		
<70 mg/dL	n/a	>85% within ± 15 mg/dL >98% within ± 40 mg/dL
70–180/dL	n/a	>70% within ± 15% >99% within ± 40%
>180 mg/dL	n/a	>80% within ± 15% >99% within ± 40%
**ADDITIONAL REQUIREMENTS**		
	≥99% within consensus error grid zones A and B	≤1% of glucose rates of change >1 mg/dL/min if true rate of change is < −2 mg/dl/min ≤ 1% of glucose rates of change < −1 mg/dL/min if true rate of change is >2 mg/dL/min

*Adapted from Freckmann et al. (56)*.

With improved accuracy some systems have received indications for non-adjunctive dosing. Devices with this distinction allow the user to make treatment decisions for both hypo- and hyperglycemia based on the CGM sensor glucose value without a confirmatory fingerstick BG measurement. These indications were granted based on the results of *in silico* modeling demonstrating the safety of this practice for systems with a MARD <10% (58). Studies support the safety of using CGM devices approved for non-adjunctive treatment decisions while also making diabetes management easier for patients (59, 60).

### Interfering Conditions and Substances

The method used to measure interstitial glucose determines the interfering substances that may impact the specific system. Literature in this area is sparse and as the use of continuous glucose monitoring expands, further exploration of medications that may affect CGM accuracy will be important.

The Dexcom, Medtronic Guardian 3, and Freestyle Libre CGM systems all make use of an enzymatic reaction between glucose and glucose oxidase to detect changes in interstitial glucose levels. Both oral and intravenous acetaminophen cause falsely elevated sensor glucose readings in older generations of sensors that use glucose oxidase, including the Dexcom G4 and G5 and the Medtronic Guardian 3. However, the addition of an acetaminophen blocker to the Dexcom G6 sensor now prevents this interference from occurring at recommended doses of acetaminophen (up to a maximum dose of 1,000 mg every 6 h) (61, 62). To the best of our knowledge, the impact of acetaminophen on glucose values measured by Dexcom G6 has only been studied using oral administration at standard doses. Higher peak serum levels are attained with intravenous acetaminophen, which may falsely elevate sensor glucose readings (63), Aspirin causes falsely low sensor glucose values in patients using the Freestyle Libre, whereas vitamin C can cause falsely high values.

In contrast to systems using glucose oxidase based measurements, the implantable Eversense sensor is coated in a fluorescent glucose-indicating polymer that emits light in a magnitude proportional to the amount of glucose present. Tetracycline causes falsely low sensor glucose values at therapeutic levels with the Eversense system; mannitol results in falsely elevated sensor glucose values when given intravenously, used for peritoneal dialysis, or for local irrigation at the sensor site (64).

As discussed previously, the accuracy of BGM, which often uses glucose oxidase for measurement, is impacted by altitude. Altitude's influence on CGM accuracy has been less well-studied; however, limited studies have suggested that extreme environments with both hypo- and hyperbaric conditions do not significantly alter the accuracy of CGM systems (33, 65, 66).

Users and clinicians should also be aware of a positional phenomenon known as compression artifact or compression hypoglycemia. Direct external pressure at the CGM sensor site results in decreased local tissue perfusion which can result in a falsely low sensor glucose value (67, 68). Compression artifact tends to occur more frequently overnight and resolves quickly with a change in position that alleviates the pressure on the sensor site (69, 70). These findings underscore the importance of being mindful of CGM sensor positioning on the body and the value of performing a confirmatory BG measurement with a glucose meter if compression hypoglycemia is suspected.

Although CGM has not been formally approved for inpatient use at this time, several studies have explored the impact of hypoperfusion, acidosis, and anemia on CGM accuracy. A study exploring the accuracy of the Medtronic Guardian in 38 pediatric patients did not find any significant effect of these metabolic perturbations on CGM accuracy (71). Another study exploring the accuracy of continuous glucose monitoring in 14 critically ill pediatric patients found that 98% of all CGM values fell in zone A or B of the Clarke error grid (72); however, a correlation between increasing MARD and acidosis and therapeutic hypothermia was observed. Despite these concerns, it should be noted that the HALF-PINT study group used CGM data to successfully titrate insulin delivery in a multi-center study exploring the impact of tight glycemic control (80–110 mg/dL) (4.4–6.1 mmol/L) on ICU-free days in critically ill pediatric patients requiring vasopressor support and mechanical ventilation (73).

### Glycemic Targets and Continuous Glucose Monitoring Systems

A1c has been the gold standard for monitoring long-term glycemic control since the Diabetes Control and Complications Trial (DCCT) showed that intensive diabetes management that decreased A1c to ~7% significantly decreased the progression or development of long-term microvascular complications in adolescents and adults with T1D (4). A recent study has shown that 10–14 days of CGM data can provide a reliable estimate of CGM metrics for the prior 3-month period (74). As a result, CGM data have been used to generate what had previously been referred to as an estimated A1c and now is referred to as the glucose management indicator (GMI) (75), which can be reported to patients and clinicians.

While A1c provides an estimate of average blood glucose values over the preceding 2–3 months, it does not provide information about glycemic variability, which may also have a role in the development of long-term diabetes complications (76, 77). Furthermore, African American race, hemoglobinopathies (78), anemia, and renal failure all affect A1c measurements (79, 80). A recent study has shown that there is considerable variability of the mean glucose level captured by the 95% confidence interval for any given A1c in individuals without any of the known confounders (81). As the use of continuous glucose monitoring increases and given these limitations of A1c, there is a movement to adopt CGM metrics as the gold standard for assessment of glycemic control (82). Beck and colleagues recently re-analyzed data from the DCCT in order to correlate the data obtained by BGM with 7 fingerstick samples per day with percentage of time in range (%TIR), so as to determine the relation between %TIR and the A1c values associated with microvascular complications (83). A recent analysis of studies reporting paired A1c and %TIR showed an excellent correlation between the two; for every 10% absolute change in %TIR, there was a 0.8% change in A1c. Based on these observations showing good correlations between A1c and %TIR, it has been suggested that a transition to %TIR should be the preferred metric for determining the outcome of clinical trials, predicting the risk of diabetes complications, and assessing individual patient's glycemic control (84).

**Figure 1:**
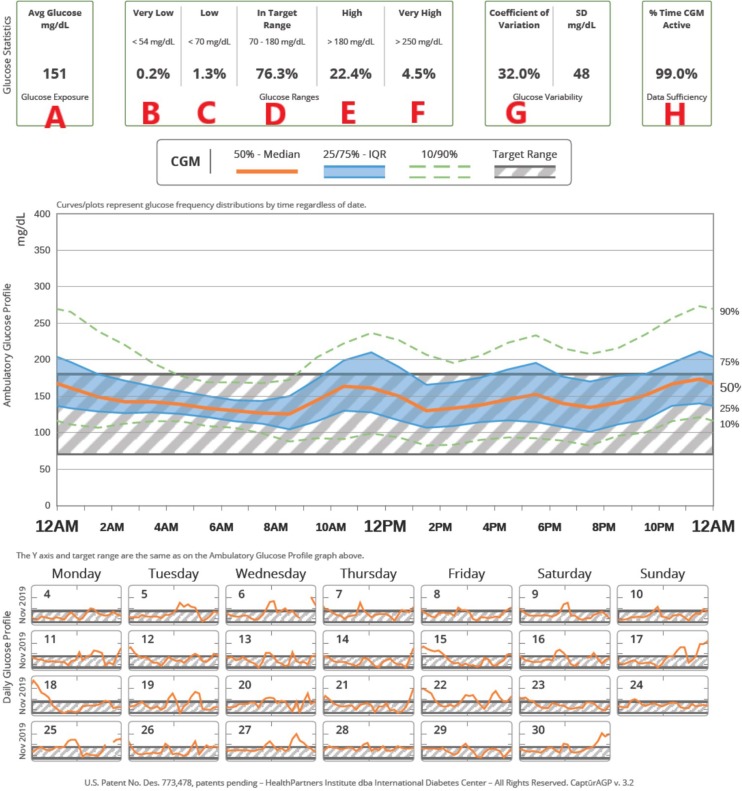
An example of how CGM metrics are displayed in a standardized ambulatory glucose profile (AGP). **(A)** Average glucose, **(B)** Time very low (<54 mg/dL), **(C)** Time low (<70 mg/dL), **(D)** Time in target range (70–180 mg/dL), **(E)** Time high (>180 mg/dL), **(F)** Time very high (> 250 mg/dL), **(G)** Coefficient of variation, **(H)** Percentage of time CGM is active. In the top figure, the hatched area shows the target range; composite data for 14-days are shown as median (orange line), interquartile range (blue shaded area), 10 and 90th percentiles (green dashed line). Individual days are shown below.

Given these efforts to move toward CGM metrics as the gold standard for assessment of glycemic control, an expert panel was convened to reach an international consensus on TIR targets (82). See Table 8 for details. The target of 70% TIR, as defined by a sensor glucose of 70–180 mg/dL (3.9–10.0 mmol/L), can be applied to patients of all ages with type 1 and type 2 diabetes. While the ISPAD guidelines recommend an A1c <7.0%, the ADA goal for pediatric patients is <7.5% (6, 7). A 60% TIR correlates with an A1c of 7.5%. The guidelines recommend less stringent targets in patients who are unable to communicate their symptoms or who have hypoglycemia unawareness, recurrent severe hypoglycemia, lack of access to insulin analogs and advanced insulin delivery technology, or inability to regularly monitor BG levels (6, 7, 82). While it is well-recognized that attaining these targets is challenging for many patients, nonetheless, the potential benefit of small improvements should be emphasized: a 5% increase in TIR has been linked to significant improvements in overall glycemic control (84, 85). The expert consensus panel also recommended that additional CGM metrics for clinical care be standardized in the ambulatory glucose profile (AGP), including: the number of days the CGM was worn (goal 14/14 days), mean glucose, percentage of time >180 mg/dL (10.0 mmol/L) (goal <25%), percentage of time >250 mg/dL (13.9 mmol/L) (goal <5%), percentage of time <70 mg/dL (<3.9 mmol/L) (goal <4%), percentage of time <54 mg/dL (3.0 mmol/L) (goal <1%), and the coefficient of variation, a measure of glycemic variability (goal ≤ 36%). Figure 1 shows an example of a standardized AGP.

**Table 8:** International consensus for clinical CGM targets.

**mg/dL**	**mmol/L**	**Percentage of time**	**Time (hours, min)**
≥250	≥13.9	<5%	72 min
>180	≥10.0	<25%	<6 h
70–180	3.9–10.0	>70%^*^	16 h, 48 min
<70	<3.9	<4%	<58 min
<54	<3.0	<1%	<15 min

* *If <25 years old with an A1c goal of 7.5% may use a target of 60%*.

### Impact of Continuous Glucose Monitoring

#### Increasing Use of CGM Over Time

Improvements in the accuracy and usability of CGM, better insurance coverage, and greater acceptance by both clinicians and patients have led to dramatic increases in the use of this technology over the past decade (86–95). In the German and Austrian population-based DPV registry, 38% of all patients were using CGMs in 2017 as compared to 3% in 2006 and 17% in 2016. The highest rates (58%) of CGM use were seen in preschool aged children (96). In 2011, just 6% of all patients in the United States Type 1 Diabetes Exchange (T1DX) registry were using CGMs as compared to 27% in the period from 2016 to 2018 (91). Again, the highest rate of CGM use was seen in young children with 51% of patients under 6 years using CGMs, as compared to the lowest rates of use in adolescents and young adults with only 22% of 18–26 years olds using the technology.

#### Disparities in Technology Use and Access

Marked healthcare disparities persist in access to and use of CGMs. Wong et al. found that CGM use was more likely to be used among T1DX participants with higher education levels, higher household incomes, and private insurance (87). These previously described differences in CGM access by race and ethnicity have persisted in the most recent analysis of the T1DX data from 2016 to 2018 (91). While non-Hispanic white children with T1D in the US are more likely to come from higher income families and to have private insurance, the disparities in access to CGM persist even after controlling for these factors. This trend suggests that there may be clinician bias in prescribing this technology despite evidence from an RCT that CGM use is equally beneficial for children of different racial and ethnic groups (97).

#### CGMs and Glycemic Control

Analysis of the T1DX data has also highlighted the impact of CGM use on glycemic control: among patients <13 years of age, the average A1c in participants using injection therapy and BGM was 9.0% compared to 8.0% in those using injections and CGMs, and 7.9% in those using insulin pumps and CGMs (91). Similar trends were seen in adolescents 13–18 years of age although the mean A1c levels were higher in all 3 groups: 9.6% in those using injections and BGM, 8.8% in those using injections and CGMs, and 8.3% in those using pumps and CGMs.

In addition to reductions in A1c, the increasing use of CGMs has been associated with decreased hypoglycemia and severe hypoglycemia (98, 99). This finding is particularly notable as improvements in A1c in the DCCT came at the expense of increased rates of severe hypoglycemia (4). Importantly, regular use of CGMs with ≥ 6 days of wear per week is essential for attaining these improved clinical outcomes (97, 99).

#### CGM Use and Patient Related Outcomes

Early studies using less accurate CGMs suggested that continuous glucose monitoring had minimal positive impact on quality of life (QoL) and, possibly, even a negative impact attributable to sleep disruption and an increased need to support those providing childcare (98, 100). However, Mauras et al. studied use of CGMs in children aged 4 to <10 years and found that CGM use is associated with increased parental satisfaction and feelings of safety (101). Randomized controlled trials of adults with T1D have shown that use of newer CGM devices positively impacts QoL by reducing diabetes distress, increasing confidence to detect hypoglycemia, and decreasing diabetes management related interpersonal tension with family and friends (102). A recently published study of a pediatric population similarly showed reduced fear of hypoglycemia, improved diabetes treatment satisfaction, and improved parental sleep quality (103).

Physical discomfort, body image concerns, the need to be connected to the device at all times, frequent alarms that disrupt daily life, and the expense of the technology are all frequently cited patient concerns regarding CGM use (44, 104–107). Parents of young children with T1D indicate that they would like to receive targeted formal education to improve their knowledge of these technologies, thereby reducing the burden of diabetes management (108). Adolescents with more depressive symptoms and lower overall and diabetes-specific quality of life are less likely to use CGMs suggesting that recognition of these factors and appropriate intervention may improve device uptake in this population (109). Further research exploring the psychosocial impact of CGM use is needed to better understand current barriers and limitations to use of CGMs in an effort to overcome barriers and increase uptake (110).

Problems with adhesion can impact wear time and may be a source of great frustration, particularly given the cost of these devices. Patients are often advised to avoid placing a CGM sensor immediately after bathing and to consider the potential for trauma and friction when selecting an insertion site. Strategies to optimize adhesion vary from one patient to another, often requiring trial and error (111). Liquid adhesive agents are often helpful and can be supplemented with adhesive patches and tape over top of the CGM sensor. Skin integrity is also a significant concern, particularly for people with sensitive skin and those who also use an insulin pump (111). Although there is little evidence-based research to guide recommendations for preserving skin integrity, current literature recommends: rotating sites to give previously used sites time to heal completely before a new sensor is inserted, and using a barrier solution or dressing (e.g., Tegaderm™) between the skin and the sensor adhesive to minimize contact and irritation.

Table 9 shows an overview of the benefits and limitations of continuous glucose monitoring.

**Table 9:** Benefits and limitations of continuous glucose monitoring.

**Benefits**	**Limitations**
• Awareness of glycemic values and trends • Alerts for impending hypo- and hyperglycemia • Use of trend arrows to optimize insulin dosing • Reduced diabetes distress • Decreased conflict with family and friends through share/ follow function • Reduced fear of hypoglycemia • Improved diabetes treatment satisfaction • Improved parental sleep • Improved glycemic control • Decreased hypoglycemia • Greater understanding of glycemic control than offered by A1c • Alerts for hypo- and hyperglycemia • Use of an algorithm to integrate CGM data with automated insulin delivery	• Body image concerns • Skin complications • Expense • Alarm fatigue • Overwhelming amount of data • Worsening healthcare disparities • Potential for technology failure • Potential for increased conflict between parents and adolescents • Lag time • Compression hypoglycemia • Interfering substances

#### Future Directions in Continuous Glucose Monitoring

Factors limiting CGM uptake among patients are areas targeted for future develpoments in continuous glucose monitoring technologies. The on body footprint of these devices remains one of the greatest concerns among adults with T1D (105). Industry is currently developing smaller devices with longer wear times with the potential for direct transmission of CGM data from the transmitter to a smart watch without the need for a nearby smart phone to serve as a receiver. Others are seeking to develop devices that combine insulin infusion cannulas with CGM sensors into a single device. Current product labeling prohibits insulin delivery at the CGM sensor site and recent studies have identified insulin preservatives as the major cause of CGM inaccuracy when these devices are in close proximity (112). While understanding current limiting factors paves a path forward for future research and development, there is also a need to prolong insulin infusion site wear time beyond the recommended 2–3 days in order to match CGM sensor wear time (113).

### Ketone Monitoring

The rate of production of ketone bodies is increased during starvation, consumption of very low carbohydrate diets, prolonged strenuous exercise, and in individuals with uncontrolled diabetes. In the liver, ß-oxidation of free fatty acids released from adipose tissue produces acetoacetic acid, which is reduced to beta-hydroxybutyric acid (BOHB), thereby regenerating NAD^+^. Acetone is produced by non-enzymatic decarboxylation of acetoacetate and is formed in relatively small amounts compared with acetoacetate and BOHB. The term ketone bodies refers to acetoacetate, BOHB and acetone. During resolution of ketosis and ketoacidosis, BOHB is first converted back into acetoacetate and then, in a series of biochemical reactions, eventually yields acetyl CoA for oxidation in the Krebs cycle.

Monitoring for the presence and severity of ketosis in appropriate circumstances is an essential component of the care of patients with T1D. See Table 10 for indications to check ketones. Ketone monitoring (based on the nitroprusside reaction) can be performed using urine test strips that measure acetoacetate and acetone. Urine test strips individually wrapped in foil to prevent exposure to air are recommended to ensure accuracy. Alternatively, blood BOHB concentration can be measured with specific meters available for use at home (in USA, Precision Xtra or Nova Max® Plus; in Europe, Abbott FreeStyle Optium, FreeStyle Optium Neo, and Menarini GlucoMen LX Plus). These devices can also measure BG using different test strips.

**Table 10:** When to measure urine or blood ketones.

•When BG is unexpectedly high; e.g., fasting ≥250 mg/dL or unexpectedly ≥300 mg/dL for more than 2–3 h • During intercurrent illness irrespective of BG concentration • With nausea, vomiting, abdominal pain • Patients using SGLT inhibitors whenever they experience malaise or nausea despite normal or only mildly elevated BG levels • Patients using an insulin pump whenever there is unexplained hyperglycemia, which may indicate failure of insulin delivery

Urine ketone monitoring is semi-quantitative; after carefully timing the reaction (15 s), the result is determined by comparing the color on the test strip with a color code on the strip container. Results may be negative, trace (5 mg/dL), small (15 mg/dL), moderate (40 mg/dL), or large (80–160 mg/dL). False negative readings may occur when strips have been exposed to air or when urine is highly acidic (e.g., after consumption of large doses of ascorbic acid). Urine ketone tests can give false positive results in patients who take valproic acid or any sulfhydryl-containing drugs, including captopril. The increasing popularity of very low carbohydrate diets for T1D management has raised new questions about baseline ketone levels in patients who adhere to these diets (114). Urine ketone testing is inexpensive and simple to perform; however, obtaining a urine sample may be challenging for parents of infants and young children, especially during an intercurrent illness. Blood BOHB levels have been correlated with urine ketone levels (Table 11). However, it should be appreciated that when an illness causes dehydration, the urine ketone measurement may be “moderate” or “large” in a concentrated urine specimen, whereas the concomitant blood BOHB level is only mildly increased.

**Table 11:** Measurement of ketosis with urine and blood ketone monitoring.

	**Urine**	**Blood**
Negative	<5 mg/dL	<0.3 mmol/L
Trace	5 mg/dL	0.3–0.5 mmol/L
Small	15 mg/dL	0.6–0.9 mmol/L
Moderate	40 mg/dL	1.0–1.5 mmol/L
Large	80–160 mg/dL	≥1.6 mmol/L

Measurement of blood BOHB concentration is quantitative and accurate up to ~5 mmol/L; however, blood ketone strips are considerably more expensive than urine ketone strips. Nonetheless, when compared to urine ketone testing, measurement of blood BOHB concentration may be cost-effective because it provides quantitative information that frequently allows parents (with guidance from their diabetes care team) to more confidently manage a sick child at home obviating the need to bring the patient to an emergency department for evaluation and treatment (115). Blood BOHB monitoring at home also offers the advantage of accurately assessing biochemical improvement after providing supplemental insulin (116, 117). When cost is a consideration, a recommended approach is to reserve blood ketone measurements for young children who cannot reliably provide a urine sample on demand and in patients who have “large” urine ketones. It is important to appreciate that mild fasting ketosis before breakfast may occur in young children with T1D in the absence of illness or metabolic deterioration (118).

The urine nitroprusside test measures acetoacetate and acetone. During ketone metabolism, BOHB is converted back into acetoacetate, which can result in prolonged ketonuria even after significant ketosis has already responded to treatment and serum BOHB has decreased to normal levels. Prolonged ketonuria can also be exacerbated by long periods of time between voiding and accumulation of older urine with a higher concentration of ketones in the bladder. Persistent ketonuria after resolution of hyperketonemia can lead to unnecessary additional insulin administration as the result of a misguided desire to rapidly “clear” the urine ketones and can cause hypoglycemia. Monitoring blood BOHB concentrations obviates this problem as its concentration reflects the rate of ketogenesis and predictably decreases in response to effective insulin therapy.

Although currently not approved for use in children and adolescents with T1D, sodium-glucose cotransporter (SGLT) inhibitors are associated with an increased risk of diabetic ketoacidosis (DKA) with only mild hyperglycemia or no hyperglycemia (euglycemic ketoacidosis). Preventing DKA in patients taking these medications relies on patients measuring blood or urine ketone levels whenever nausea, vomiting, abdominal pain or lethargy occurs (119).

## Conclusions

In conclusion, regular monitoring of BG and ketone levels, in appropriate circumstances, is essential for optimal management of pediatric T1D. Frequent BGM improves A1c while also decreasing hypo- and hyperglycemia. Whereas, BGM has been the cornerstone of T1D management for decades, with increasing accuracy, cost-effectiveness and acceptance of CGM, this technology is expected to become the universal standard of care in the near future. A thorough understanding of CGM technology, including factors affecting its accuracy, is critical in order for clinicians to thoroughly educate and guide patients in its use. It is important to recognize that CGM has limitations and can fail; therefore, BGM must be available for backup and must remain an essential component of comprehensive T1D care. Monitoring of urine or blood ketone levels when indicated, coupled with BGM and appropriate intervention, can often prevent progression from ketosis to ketoacidosis and obviate the need for care in an emergency department and admission to hospital.

## Author Contributions

BM and JW contributed equally to the literature review and writing of this article and accountable for the content.

### Conflict of Interest

The authors declare that the research was conducted in the absence of any commercial or financial relationships that could be construed as a potential conflict of interest.
